# How agriculture, manufacture, and urbanization induced carbon emission? The case of Indonesia

**DOI:** 10.1007/s11356-020-10148-w

**Published:** 2020-07-24

**Authors:** Slamet Eko Prastiyo, Irham, Suhatmini Hardyastuti, Jamhari

**Affiliations:** 1 Central Java Provincial Government: Agriculture and Plantation Services, Kompleks Tarubudaya, Ungaran, Jawa Tengah Indonesia; 2 Faculty of Agriculture, Universitas Gadjah Mada, JL. Flora, Bulaksumur, Yogyakarta, 55281 Indonesia

**Keywords:** Agriculture, Causality, Environmental Kuznets Curve (EKC), emission, Manufacture, Urbanization

## Abstract

The agriculture and manufacturing sectors are the backbones of the Indonesian economy; for this reason, research on the effects of these sectors on carbon emissions is an important subject. This work adds urbanization to enrich research on the Environmental Kuznets Curve (EKC) in Indonesia. The results of this study indicate that the EKC hypothesis was confirmed in Indonesia with a turning point of 2057.89 USD/capita. The research results show that all variables affect the escalation of greenhouse gas emissions in Indonesia. Furthermore, there is a bidirectional causality relationship between emissions with economic growth, emissions with agricultural sector, emissions with manufacturing sector, economic growth with agricultural sector, and economic growth with manufacturing. The unidirectional causality is found in emissions by urbanization and economic growth by urbanization. To reduce the impact of environmental damage caused by the activities of agriculture, manufacturing, and urbanization sectors, it is recommended that the government conduct water-efficient rice cultivation and increase the use of renewable energy.

## Introduction

Economic growth and human activities have caused an increased concentration of greenhouse gas (GHG) emissions in the atmosphere. Carbon dioxide concentrations have increased by 40% since pre-industrial times, mainly from fossil fuel emissions and also from land use including the agricultural sector (IPCC 2013). One of the main GHG emitters in the world is the agriculture sector, which accounts for at least 20% of total emissions worldwide, of which more than 44% of agricultural sector emissions are generated in the Asian continent (FAO 2016a, b). Meanwhile, the industry contributes directly and indirectly to about 37% of the global greenhouse gas emissions. Total energy–related industrial emissions have grown by 65% since 1971 (Worrell et al. 2009). Economic growth in the industrial sector, especially manufacturing and construction, not only results in increasing the welfare of the community but also triggers environmental damage, including through GHG emissions. In the manufacturing sector, GHG emissions are generated through the use of chemicals and fuels in industrial processes (Tan et al. 2011; Peng et al. 2012; Mi et al. 2015; Asghar et al. 2019; Zaekhan et al. 2019). The growth of the industry also causes an increase in the flow of urbanization, but a unique two-way causality relationship also occurs where urbanization can also increase economic and industrial development (Cheng 2013; Xia et al. 2017; Nguyen and Nguyen 2018). Meanwhile, urbanization has also led to the use of fuels such as electricity, oil, natural gas, and coal thereby increasing GHG emissions in the earth’s atmosphere (Brown 2012; Kurniawan and Managi 2018).

This study emphasizes how the influence of the agriculture, manufacturing, and urbanization sectors on greenhouse gas (GHG) emissions according to the hypothesis of the Environmental Kuznets Curve (EKC) in Indonesia. This study is highly relevant and greatly contribute partly because the agriculture and manufacturing sectors have been the backbone of the economy in Indonesia. The value of agriculture value added in the agricultural sector continues to grow from 23.57 billion USD in 1960 to 143.78 billion USD in 2018. However, along with the increasing growth of the industrial and service sectors, the contribution of the agricultural sector to GDP continues to decline. In 1960, the contribution of the agricultural sector to GDP was 34.22%, down to only 12.54% in 2018 (World Bank 2019). Although the contribution of the agricultural sector continues to decline, however, until 2018, the agricultural sector still occupies the second largest sector that underpins Indonesia’s economic growth just below the manufacturing sector (BPS 2019). Meanwhile, the manufacturing sector grew rapidly in Indonesia, where the manufacturing value added in Indonesia was only 4.37 billion USD in 1960 which increased to 241.27 billion USD in 2018. The contribution of the manufacturing sector to GDP also increased, where in 1960, its contribution was only around 7.73% and it has now risen to 21.04% in 2018 (World Bank 2019). Considering the large contribution of the manufacturing and agricultural sectors, i.e., up to 33% of GDP, there is no doubt that both sectors are vital sectors for the Indonesian economy and at the same time high contribution to GHG emissions.

Industrial growth, especially the manufacturing sector, is driving urbanization, especially in developing countries (Gollin et al. 2016; Nguyen and Nguyen 2018). Various studies show that industrialization and urbanization increase the intensity of energy use including in Indonesia (Sadorsky 2013; Kurniawan and Managi 2018). This manuscript provides a novelty in, that is, knowing how the influence of vital sectors, namely agriculture, manufacturing, and urbanization, toward GHG emissions for developing countries such as Indonesia. Although the EKC hypothesis has been widely used in prior research, however, the simultaneous use of both variables agriculture and manufacturing sector in the EKC hypothesis has never been carried out in earlier studies.

## Literature review

The study of EKC was first used by Grossman and Krueger (1991) which was very useful to describe the relationship between economic growth and environmental damage. Along with increasing global awareness about climate change and global warming, the EKC hypothesis with the GHG emission variables is applied to study environmental damage. Various EKC studies using GHG variables were carried out in the single country (Khan et al. 2019; Sasana and Aminata 2019; Shujah-ur-Rahman et al. 2019; Usman et al. 2019) or multiple countries (Rauf et al. 2018; Balsalobre-lorente et al. 2019; Elshimy and El-Aasar 2019; Zhang 2019)

Research on EKC then developed further by using various variables as proxies, including energy (Destek and Sarkodie 2019; Hundie and Daksa 2019; Usman et al. 2019), financial sector development (Tamazian and Rao 2010; Charfeddine and Ben 2016; Aye and Edoja 2017), government performance (regulation, corruption index, education index) (Leitão 2010; Castiglione et al. 2012; Rehman et al. 2012; Zhang et al. 2016; Chen et al. 2018), foreign direct investment (Cole et al. 2011; Sapkota and Bastola 2017), and trade variables (Ertugul et al. 2016; Ozatac et al. 2017; Park et al. 2018; Twerefou et al. 2019).

Although the agriculture and manufacturing sectors are the main drivers of economic growth in various countries (Szirmai and Verspagen 2015; Junankar 2016; McArthur and McCord 2017), research on the EKC hypothesis by using combined agriculture and manufacturing sectors as variables has not been a priority for researchers so far. EKC research with agricultural sector variables as exogenous variables, among others, was conducted by Qiao et al. (2019) in G20 countries and Gokmenoglu and Taspinar (2018) in Pakistan. The results showed that the agricultural sector has a positive effect on increasing GHG emissions, while the different results that are shown by the research including Liu et al. (2017) in 4 ASEAN countries (Indonesia, Malaysia, the Philippines, and Thailand); Rafiq et al. (2016) in 53 countries, namely 30 low-medium income countries and 20 megara high income; Dogan (2016) in Turkey; Jebli and Youssef 2017) in northern Africa; Mamun et al. (2014) researchers in 136 countries; and Nugraha and Osman (2018) in Indonesia show that the agricultural sector has instead a negative influence on GHG emissions.

This study also uses a manufacturing sector variable where the sector produces emissions through the use of fuels and chemicals in its process (Tan et al. 2011; Peng et al. 2012). Research on the EKC hypothesis by using a manufacturing sector proxy is rarely conducted. Research from Zhang et al. (2019) about the EKC hypothesis using manufacturing and industrial sector emissions in 121 countries shows that the EKC hypothesis is proven in 95 countries. Moreover, further research from Ahmad et al. (2019) showed that in China’s construction sector, it concluded the EKC hypothesis and played a significant role in increasing GHG emissions. EKC research with industrial sector proxies (including manufacturing) was carried out among others by Asghar et al. (2019) in 13 Asian countries; Hao et al. (2016) in China; Luo et al. (2017) in G20 countries; and Nguyen et al. (2019b) in emerging economies. In all of these publications, it was concluded that the industry has positive effects on GHG emissions. The study from Ren et al. (2014) concluded that the per capita income of the industrial sector led to increased CO_2_ emissions. Different results obtained by Xu and Lin (2017) indicated that the high-tech industry will in the long run reduce the level of GHG emissions in China. The ability of high-tech industries to reduce emissions is due to the greater use of renewable energy in the high-tech industry.

Urbanization that is driven by economic growth and industrialization is driving the increasing use of fuels that cause GHG emissions (Xia et al. 2017; Kurniawan and Managi 2018; Nguyen and Nguyen 2018). Urbanization is also one of the proxy variables used in various studies of the EKC hypothesis. The results of the majority of studies show that urbanization is a driver of environmental damage. Research concluding that urbanization has a positive effect on increasing GHG emissions, among others, is carried out by Aung et al. (2017) in Myanmar; Hao et al. (2016), Kang et al. (2016), Li et al. (2016), and Ahmad et al. (2019) in China; Khan et al. (2019) in Pakistan; Al-Mulali et al. (2016) in Kenya; Ozatac et al. (2017) and Pata (2018) in Turkey; Dogan and Turkekul (2016) in the USA; Shahbaz et al. (2014) in United Arab Emirates; Kasman and Duman (2015) in new EU members and candidate countries; Al-mulali et al. (2014) in 93 countries; and Zhang et al. (2019) in Central Asian countries, while different conclusions are generated by research from Nguyen et al. (2019b) in 33 emerging economies where urbanization has a positive effect on emissions from the manufacturing and settlement sectors; however, negative effect on emissions from commercial buildings and transportation was observed. Research of Nguyen et al. (2019a) in 106 countries concludes that urbanization increases CO_2_ and N_2_O (nitrous oxide) gas emissions but has no effect on CH_4_ (methane) gas. The research from Azam and Qayyum (2016) shows that urbanization has a negative effect in the USA but has an insignificant effect in China, Tanzania, and Guatemala. On the contrary, research from Diputra and Baek (2018) concluded that urbanization had no significant effect on emissions in Indonesia.

Earlier studies on the EKC hypothesis in Indonesia have been conducted, among others, by Alam et al. (2016) and concluded that the EKC hypothesis with exogenous variables of energy consumption and population growth contributed positively to CO_2_ emissions. Other research by Sugiawan and Managi (2016) concluded that the EKC hypothesis with exogenous variables renewable energy can reduce emissions; while the research of Saboori et al. (2012), Liu et al. (2017), and Sasana and Aminata (2019) concluded that the EKC hypothesis did not occur in Indonesia. However, the research conducted by Waluyo and Terawaki (2016) concluded the EKC hypothesis occurred in Indonesia.

## Methodology

### Data

The aim of this research is to investigate the EKC hypothesis in Indonesia and its influence from the agriculture, manufacturing, and urbanization sectors. The causality relationship between variables is also the focus of this study. Data in the form of annual data from 1970 to 2015 were the source of the data obtained from World Bank (2019) and EDGAR (2019). The data used are GHG emissions from EDGAR, while gross domestic products as a proxy of economic growth; Agriculture Value Added, Manufacturing Value Added, and Urbanization were obtained from the World Bank.

### Model

The goal of this study is to find out whether the agriculture, manufacturing sector, and urbanization sectors cause GHG emissions under the EKC hypothesis. Study of the development of the EKC hypothesis was done by investigating the agricultural sector regarded as a regressor factor by Jebli and Youssef (2017), urbanization by Zhang et al. (2019), industry (including manufacturing and construction) (Xu and Lin 2017; Zhang et al. 2019), and urbanization along with construction (Ahmad et al. 2019). Previous studies proved that agriculture, industry, and urbanization affect greenhouse gas emissions. So the equation from this research is

1 $$ {\mathrm{CC}}_t=f\ \left({\mathrm{GDP}}_t,{{\mathrm{GDP}}^2}_t,{\mathrm{Ava}}_t,{\mathrm{MG}}_t,{\mathrm{Urb}}_t\right) $$

Equation (1) is changed to Eq. (2) and to check the EKC hypothesis:

2 $$ {\mathrm{CC}}_t={\alpha}_0+{\alpha}_1{\mathrm{GDP}}_t+{\alpha}_2{\mathrm{GDP}}_t^2+{\alpha}_3{\mathrm{Ava}}_t+{\alpha}_4{\mathrm{MG}}_t+{\alpha}_5{\mathrm{Urb}}_t+{\mu}_t $$

where the definition and expected sign of each variable are presented in Table 1.

**Table 1:** Variable definitions

Variable	Definition	Unit	Source	Expected signs	Expected signs based on research
CC	Total carbon emissions per capita.	ton CO_2_eq/capita	EDGAR		
GDP	Gross domestic product per capita	USD (constan 2010)/capita	Worldbank	+	Dinda (2004)
GDP^2^	Gross domestic product per capita square	(USD (constan 2010)/capita)^2^	Worldbank	-	Dinda (2004)
Ava	Percentage of agriculture value added/GDP	%	Worldbank	-	Jebli and Youssef (2017); Liu et al. (2017); Nugraha and Osman (2018)
MG	Percentage of manufacturing value added/GDP	%	Worldbank	+	Ahmad et al. (2019); Asghar et al. (2019)
URB	Percentage of urban population to the total population	%	Worldbank	+	Ahmad et al. (2019); Zhang et al. (2019)

Manufacturing refers to industries belonging to ISIC (The International Standard Industrial Classification of All Economic Activities) divisions 15–37. Value added is the net output of a sector after adding up all outputs and subtracting intermediate inputs (World Bank 2019)

In general, the estimation model to test the significance of the coefficient *α* for the purposes of the EKC hypothesis according to Dinda (2004) is as follows:

- If *α*_1_ = *α*_2_ = 0, then there is no relationship between *x* and *y*
- If *α*1 > 0 and *α*2 = 0, a linear and increasing relationship exists between *x* and *y*
- If *α*1 < 0 and *α*2 = 0, a linear and decreasing relationship exists between *x* and *y*
- If *α*1 > 0, *α*2 < 0 , there is an inverse U relationship between *x* and *y*, so EKC occurs
- If *α*1 < 0, *α*2 > 0 , U-shape curve occurs

where the turning point of the EKC hypothesis curve is as follows: $$ \mathrm{GDP}=\left(\raisebox{1ex}{$-{\alpha}_1$}\!\left/ \!\raisebox{-1ex}{$2{\alpha}_2$}\right.\right) $$

### Bound test cointegration

The bound test cointegration test was performed using the autoregressive distributed lag (ARDL) method (Pesaran and Shin 1999; Pesaran et al. 2001; Narayan 2005). The use of ARDL is caused because first, this approach does not impose conditions that the variables have the same integration sequence. However, stationary variables that are integrated into order 1 or order 0 should be taken into account. Second, ARDLs correspond to a small sample. Third, in the ARDL model, the dependent variable is explained by the past and by the past of other independent variables (Cherni and Jounini 2017)

The requirement for variables to be used in the ARDL model is to be stationary in order 0 or order 1 (first difference), so that a root test is performed to test stationarity. There are various methods of doing a stationary test (root test) such as the augmented Dickey Fuller (ADF) test (Dickey and Fuller 1979), Kwiatkowsky, Phillips, Schmidt and Shin (KPSS) (Kwiatkowski et al. 1992), or Phillip Perron (PP) test (Phillips and Perron 1988); however, all tests will be biased and spurious when there is a structural break in the time series data. To overcome this, Zivot and Andrews (1992) developed mathematical models to find out when there is a structural break in the data. The equation was developed by Zivot and Andrews to test the model, as used by Shahbaz et al. (2013), is as follows:

3 $$ {\Delta  x}_t=a+{ax}_{t-1}+ bt+\kern0.5em {\mathrm{cDU}}_t+{\sum}_{j=1}^k{d}_j{\Delta  x}_{t-j}+{\mu}_t $$

4 $$ {\Delta  x}_t=b+{ax}_{t-1}+ ct+{\mathrm{cDT}}_t+{\sum}_{j=1}^k{d}_j{\Delta  x}_{t-j}+{\mu}_t $$

5 $$ {\Delta  x}_t=c+{\mathrm{cx}}_{t-1}+ ct+{\mathrm{dDU}}_t+{\mathrm{dDT}}_t+{\sum}_{j=1}^k{d}_j{\Delta  x}_{t-j}+{\mu}_t $$

where the dummy variable is shown by DU_*t*_ which indicates a shift in the average value at each point with time break while the trend shift variable is indicated by DT_*t*_. So:

$$ {\mathrm{DU}}_t=\left\{\begin{array}{c}1\dots \mathrm{if}\ t>\mathrm{TB}\\ {}0\dots \mathrm{if}\ t<\mathrm{TB}\end{array}\ \mathrm{and}\ {\mathrm{DU}}_t=\left\{\begin{array}{c}t-\mathrm{TB}\dots \mathrm{if}\ t>\mathrm{TB}\\ {}0\dots \mathrm{if}\kern0.75em t<\mathrm{TB}\end{array}\right.\right. $$

The null hypothesis of the unit root break date is *c* = 0 which indicates that the data is not stationary and has no information about structural breakpoints while the hypothesis *c* < 0 implies that the variable obtained has been trend stationary with an unknown time break. The Zivot-Andrews root unit test corrects all points as a potential for possible time breaks and successfully provides estimates through regression analysis for all time breaks. Then, this unit root test selects a time break which reduces one side of the t-statistic to test $\hat{c}\left(=c-1\right)=1$. Zivot-Andrews suggests that with an endpoint, there is a distribution of asymptotic statistics from statistical deviations to infinity. It is important to choose the region where the end of the sample period is excluded.

Equation (2) is then reformulated to estimate cointegration with the ARDL model (Narayan 2005; Shahbaz et al. 2013). Cointegration means that despite being individually nonstationary, a linear combination of two or more time series can be stationary. Cointegration of two (or more) time series suggests that there is a long-run, or equilibrium, relationship between them (Gujarati and Porter 2009). Finally, the unrestricted correction error model (UECM) derived from ARDL boundary testing is used to integrate short-term dynamics with long-term balance (Pesaran and Shin 1999; Pesaran et al. 2001; Shahbaz et al. 2013). Following these lines, the UECM model for carbon emissions in Indonesia is as follows:

6 $$ \Delta  {\mathrm{CC}}_t={\alpha}_0+{\sum}_{i=1}^{\rho }{\alpha}_{1i}{\Delta  \mathrm{CC}}_{t-i}+\kern0.75em {\sum}_{i=1}^{\rho }{\upalpha}_{2i}{\Delta  \mathrm{GDP}}_{t-i}+\kern0.5em {\sum}_{i=1}^{\rho }{\alpha}_{3i}{\mathrm{GDP}}_{t-1}^2+{\sum}_{i=1}^{\rho }{\alpha}_{4i}{\Delta  \mathrm{Ava}}_{t-i}+{\sum}_{i=1}^{\rho }{\alpha}_{5i}{\Delta  \mathrm{MG}}_{t-i}+\kern0.75em {\sum}_{i=1}^{\rho }{\alpha}_{6i}{\Delta  \mathrm{Urb}}_{t-i}+{\lambda}_1\mathrm{CO}{2}_{t-1}+{\lambda}_2{\mathrm{GDP}}_{t-1}+{\lambda}_3{\mathrm{GDP}}_{t-1}^2+{\lambda}_4{\mathrm{Ava}}_{t-1}+{\lambda}_5{\mathrm{MG}}_{t-1}+{\lambda}_6{\mathrm{Urb}}_{t-1}+{\lambda}_7\mathrm{Dumbreak}+{\mu}_t\ \Big)\kern0.75em $$

where *α* is an intercept, *λ* is the long-run coefficient tested for cointegration, *t* is the period time used, *i* is lag order, and *ɛ* is a white noise error term. The optimal value of lag (*ρ*) in Eq. 6 was selected based on the Akaike information criterion (AIC). The minimum value on the AIC shows the optimal value of *ρ*. The Dum is a dummy of structural break where the value 1 is used after the break date and the value 0 is used before. The significance level of the influence of the independent variables together from the lag level in this equation is used the F-test, where the null hypothesis and the alternatives stated as follows are H0 are *α*_1_ = *α*_2_ = *α*_3_ = *α*_4_ =*α*_5_ = *α*_6_ = 0 (no cointegration) and H1 are *α*_i_ ≠ 0, *α*_2_ ≠ 0, *α*_3_ ≠ 0, *α*_4_ ≠ 0, *α*_5_ ≠ 0, *α*_6_ ≠ 0 (cointegration exists). The distribution of F-test statistical values on null hypotheses and test hypotheses is based on research conducted by Pesaran and Shin (1999) and Pesaran et al. (2001), and uses Narayan (2005). If the F-test is higher than the upper critical limit, the null hypothesis of no cointegration is rejected, regardless of whether the variable is *I* (0) or *I* (1). Conversely, when F-test is less than the lower critical limit value, the null hypothesis is not rejected, and it is concluded that there is no long-term relationship between the variables studied. However, if F-test is between the lower and upper critical values, the results cannot be concluded. Diagnostic tests such as correlation, normality, heteroscedasticity tests are carried out to ensure acceptance of the model. Besides, stability tests such as cumulative sum (CUSUM) and cumulative sum squared (CUSUMQ) are performed to see the stability parameters of the model.

### Causality

If cointegration occurs in the ARDL model, it can be concluded that there is a causal relationship in the variable, at least there is a one-way relationship. The ARDL model cannot only see the effect of the regressor variable on the dependent variable, but it cannot investigate the causality relationship either. This is important because an association or correlation between variables does not necessarily imply causation. Causality answers the question as to whether the past values of one variable (e.g., GDP) can help improve the prediction of another variable (e.g., CO_2_) aside the one provided by its own past values. Causality measures the precedence and information content of GDP for CO_2_ and vice versa (Aye and Edoja 2017). In the case of a causality test, the VECM Granger Causality is used which can detect short-term and long-term causality where the coefficient ECM_*t* − 1_shows long-term causality. The VECM Granger causality equation for each form of carbon emissions both total emissions and emissions from the agricultural and manufacturing sectors is as follows:

7 $$ \left(1-L\right)\left[\begin{array}{c}{CC}_t\\ {}{GDP}_t\\ {}{GDP^2}_t\\ {}{Ava}_t\\ {}{MG}_t\\ {}{Urb}_t\end{array}\right]=\left[\begin{array}{c}{a}_1\\ {}{a}_2\\ {}{a}_3\\ {}{a}_4\\ {}{a}_5\\ {}{a}_6\end{array}\right]+{\sum}_{i=1}^{\rho}\left(1-L\right)\left[\begin{array}{c}{b}_{11i}{b}_{12i}{b}_{13i}{b}_{14i}{b}_{15i}{b}_{16i}{b}_{17i}\\ {}{b}_{11i}{b}_{12i}{b}_{13i}{b}_{14i}{b}_{15i}{b}_{16i}{b}_{17i}\\ {}{b}_{11i}{b}_{12i}{b}_{13i}{b}_{14i}{b}_{15i}{b}_{16i}{b}_{17i}\\ {}{b}_{11i}{b}_{12i}{b}_{13i}{b}_{14i}{b}_{15i}{b}_{16i}{b}_{17i}\\ {}{b}_{11i}{b}_{12i}{b}_{13i}{b}_{14i}{b}_{15i}{b}_{16i}{b}_{17i}\\ {}{b}_{11i}{b}_{12i}{b}_{13i}{b}_{14i}{b}_{15i}{b}_{16i}{b}_{17i}\end{array}\right]\left[\begin{array}{c}{CC}_{t-1}\\ {}{GDP}_{t-1}\\ {}{GDP^2}_{t-1}\\ {}{Ava}_{t-1}\\ {}{MG}_{t-1}\\ {}{Urb}_{t-1}\end{array}\right]+\left[\begin{array}{c}{\theta}_1\\ {}{\theta}_2\\ {}{\theta}_3\\ {}{\theta}_4\\ {}{\theta}_5\\ {}{\theta}_6\end{array}\right]\left[{ECM}_{t-1}\right]+\left[\begin{array}{c}{\varepsilon}_{1t}\\ {}{\varepsilon}_{2t}\\ {}{\varepsilon}_{3t}\\ {}{\varepsilon}_{4t}\\ {}{\varepsilon}_{5t}\\ {}{\varepsilon}_{6t}\end{array}\right] $$

where *L* is the backward operator, *a* is the constant term, *ρ* is the indication of lag length, *b* is a parameter, *ECM*_*t* − 1_ is error correction term, *θ* is a coefficient *ECM*_*t* − 1_, and *ε* is a random error term. Cointegration equation values will show the error coefficient. The significant value of the first difference lagged estimation of the variable tested by the Wald test is used to obtain short-term causality, while the long-term causality is obtained from the results of the t static estimate of the value *ECM*_*t* − 1_.

## Result and discussion

### Descriptive statistics and correlation

Statistical results of the description and correlation matrix can be seen in Table 2. From the correlation matrix, it can be noticed that there is a strong correlation between emissions with GDP, GDP with Ava, GDP with MG, GDP with Urb, Ava with MG, Ava with Urb, and Mg with Urb.

**Table 2:** Descriptive statistics and correlation matrix

	CC	GDP	AVA	MG	Urb
Descriptive statistics					
Mean	32.94864	1958.051	20.04988	17.60186	34.12457
Median	32.25355	1923.695	18.14819	19.77967	33.2555
Maximum	44.62084	3824.275	34.22502	24.23369	53.313
Minimum	28.61499	772.1297	13.0414	7.703786	17.071
Std Dev	3.640471	848.1799	5.939563	5.821146	11.88815
Skewness	1.498763	0.48979	0.789762	-0.49645	0.103532
Kurtosis	5.335476	2.304025	2.591374	1.680479	1.592088
Jarque-Bera	27.67592	2.76759	5.101918	5.226753	3.881425
Probability	0.000001	0.250626	0.078007	0.073287	0.143602
Sum	1515.637	90070.36	922.2945	809.6854	1569.73
Sum Sq dev	596.3863	32373408	1587.528	1524.858	6359.765
Observations	46	46	46	46	46
Correlations					
CC	1				
GDP	− 0.7572	1			
AVA	0.2125	− 0.90523	1		
MG	− 0.4234	0.838051	−0.94934	1	
Urb	− 0.225	0.972144	− 0.92834	0.917063	1

### Root test

The stationarity test using the Zivot-Andrews breakpoint method was performed as a condition so that the ARDL model could be used. For comparison, the Phillip Perron unit root test by Phillips and Perron (1988) was also performed in this study. All variables used must be stationary either in *I* (0) or in *I* (1). Table 1 is the result of the stationarity test where all variables are stationary either in *I* (0) or in *I* (1)in both tests and produce a breakpoint year in the Zivot-Andrews test. The results of the Zivot-Andrews test on the CC variable used as the dependent variable in this study resulted a break year in 1981. This was possible because at that time Indonesia was experiencing an increase in the amount of energy used. The early 1980s was the time when Indonesia went through an increase in consumption and production due to the second oil boom period of 1979–1980. Data from British Petroleum (2019) shows that fuel consumption in Indonesia in 1981 jumped to 20.6 million tons from only 18 million tons in 1980.

### Cointegration

Table 3 shows the results of the bound test cointegration test, where the minimum AIC which is the optimal lag value is 4 so that the model is obtained (4, 3, 4, 4, 4, 1, 2). The statistical *F* value uses the bound test value from Pesaran et al. (2001) and Narayan (2005), showing that the statistical F-test is above the upper bound with a level of 1%. So it can be concluded that there was cointegration in both models.

**Table 3:** Zivot-Andrews and Phillip-Perron Stationary test

Unit Root test	Zivot-Andrews test				Phillip-Pherron test	
	Level	Break	First difference	Break	Level	First difference
Variable						
CC	− 3.7960	1982	− 4.8891***	1981	− 3.1010	− 4.3104***
GDP	− 2.2046	2008	− 5.6617***	1997	2.3915	− 4.4275***
GDP^2^	− 0.7388	2008	− 5.0923***	2007	5.2799	− 3.1102**
Ava	− 2.8515	1994	− 6.3429***	1978	− 4.140***	− 5.0063***
MG	− 1.7412	2005	− 5.9846***	1984	− 1.9281	− 3.490**
Urb	− 3.2079	1991	− 5.9345***	2001	− 3.6989**	− 2.4117

*,**,***10, 5, and 1% level of significance

**Fig. 1:**
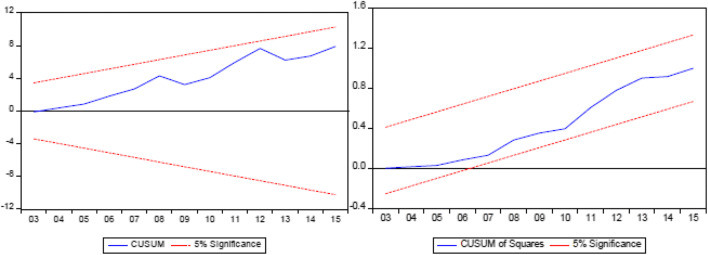
Residual stability test (Cusum and Cusum of Square)

The models were analyzed for both long- and short-term relationships and results obtained from the integration are presented in Table 4. To test the reliability of the model, normality diagnosis, serial correlation, and heteroscedasticity tests were carried out according to research from Pesaran et al. (2001). The diagnostic test results showed that the model successfully passed the tests of normality, serial correlation, and heteroscedasticity. The model stability test used cumulative number (CUSUM) and cumulative squared number (CUSUMQ) in Fig. 1 shows that the model is stable.

**Table 4:** Value of bound test cointegration

Model		Optimal lag	F-statistic	Cointegration
Model CC (4,3,4,4,4,1,2)		4	9.326673***	Yes
Bound test value	Pesaran et al. (2001)		Narayan (2005)	
	Upper bound	Lower bound	Upper bound	Lower bound
1%	4.43	3.15	5.463	3.800
5%	3.61	2.45	4.211	2.797
10%	3.23	2.12	3.599	2.353

*,**,***10, 5, and 1% level of significance

The analysis test results in Table 4 show that economic growth will worsen the quality of the environment. The test results also show that an increase in GDP/capita of 1 USD/capita will increase carbon emissions by 0.128 ton CO_2_eq/capita. The GDP2 variable in the model is negative and significant so it refers to the criteria of Dinda (2004) then there is an inverse U curve so that the Environmental Kuznets Curve exists. The occurrence of the EKC hypothesis in the model is consistent with various previous studies on EKC in Indonesia (Alam et al. 2016; Sugiawan and Managi 2016; Diputra and Baek 2018; Kurniawan and Managi 2018; Sasana and Aminata 2019; Zhang et al. 2019). The confirmation of the EKC hypothesis on the model with turning points is 2057.89 USD/capita. With a 2015 GDP of 3824.27 USD/capita, the turning points for Indonesia have been exceeded. The exceedance of the EKC proves that in the future it is expected that the level of GHG emissions per capita in Indonesia will decrease. Efforts to reduce the level of emissions have been made since Indonesia signed the Kyoto Protocol in 1998 and ratified it in 2004. More comprehensive efforts have been carried out since 2009 with the issuance on Rencana Aksi Pengurangan Emisi Gas Rumah Kaca (RAN GRK) or Decree for GHG Emission Reduction governing the plan detailed emission reductions in each sector (Endah Murniningtyas et al. 2015; Kawanishi et al. 2016). Indonesia has also developed a model of sustainable development in the energy sector, especially electricity, for a long time. The use of hydroelectric power plants has been carried out since the 1970s, the use of renewable energy for electricity and then developed by using geothermal, micro hydro, solar energy (Rozali et al. 1993; Handayani et al. 2019; Nasruddin et al. 2020). Even in 2018, the Joko Widodo administration officially used wind power for the first time for electricity in Indonesia (Hajramurni 2018). The use of renewable energy for the electric energy sector is expected to be able to reduce GHG emissions from the electricity sector by 25% (Handayani et al. 2019). In the transportation sector, it is targeted that in 2025, Indonesia will use 10.22 million kiloliters of biodiesel from palm oil (Handoko et al. 2012)

The role of the agricultural sector as a cause of carbon emissions in Indonesia is tested using the variable agriculture value added/GDP. The result of agriculture value added/GDP is negative and significant in accordance with expected signs from this study. So every 1% increase in agriculture value added/GDP will reduce carbon emissions by 2.53 ton CO_2_eq/capita. However, with the condition of the continued decline in the contribution of the agricultural sector in Indonesia, it will lead to a case in which every 1% reduction in the contribution of Ava/GDP will increase GHG emissions by 2.53 ton CO_2_eq/capita. This is consistent with the studies from Nugraha and Osman (2018) in Indonesia; Anwar et al. (2019) in low-middle income countries including Indonesia; Balsalobre-lorente et al. (2019) in Brazil, Russia, India, China, and South Africa; Dogan (2016) in Turkey; Liu et al. (2017) in 4 ASEAN countries (Indonesia, Malaysia, the Philippines, and Thailand); Rafiq et al. (2016) in 65 countries; and Asumadu-sarkodie and Owusu (2017) in Ghana. According to FAOSTAT, Indonesia’s GHG emissions in 2018 amounted to 0.165 MegatonCO_2_eq where rice cultivation is the main producer of agricultural sector GHG emissions. The calculation results similar to FAO were conducted by Hasegawa and Matsuoka (2015) where the largest GHG emitters in Indonesia were from rice cultivation by 37% of total emissions in the agricultural sector. Rice cultivation in Indonesia is the largest emitter because farmers still use subsidized chemical fertilizers (Rachman and Sudaryanto 2010; Warr and Yusuf 2014). The use of chemical fertilizers, especially in wet paddy fields for rice cultivation, has resulted in increased GHG emissions, mainly in the form of methane (CH_4_) and nitrous oxide (N_2_O) (Setyanto et al. 2000; Deangelo et al. 2006).

While the variable manufacturing value added/GDP affects the increase in carbon emissions, a 1% increase in MG will increase total emissions by 1688 ton CO_2_eq/capita. Although there is still very little research linking carbon emissions with the manufacturing sector, research with MG or similar variables with this variable is conducted among others (Zhang et al. 2019) which conducts researches in 121 countries including Indonesia, Ahmad et al. (2019) in the construction sector in China, and Asghar et al. (2019) to the industrial sector in Asia; and Nguyen et al. (2019b) in the industrial sector in emerging economies including Indonesia. The manufacturing sector in Indonesia is dominated by the food and beverage industry, the coal, oil, and gas refinery industry, the transportation equipment industry, the metal goods industry, and the chemical industry (BPS 2019). The use of chemicals and fuels in the manufacturing industry process causes increased GHG emissions, especially in the chemical, automotive, coal, oil, and gas industries and automotive (Tan et al. 2011; Peng et al. 2012; Mi et al. 2015; Asghar et al. 2019; Zaekhan et al. 2019), while a special study in Indonesia conducted by Zaekhan et al. (2019) states that the use of various types of fossil fuels and the increase in total manufacturing output as a major cause of high GHG emissions in the manufacturing sector.

Urbanization as previously thought will have an impact on the model of environmental damage. Every 1% increase in the ratio of urban population to the total population will increase emissions by 14,278 ton CO_2_eq/capita. Research results about the relationship between urbanization and carbon emissions in Indonesia produce different results. For example, research from Kurniawan and Managi (2018) shows that urbanization will increase carbon emissions in Indonesia. While research from Diputra and Baek (2018) shows that urbanization does not affect carbon emissions in Indonesia. The results of this research are similar to the findings reported by Kurniawan and Managi (2018). Furthermore, it is also consistent with various studies that investigated the relationship between urbanization and carbon emissions, among others, Ahmad et al. (2019) in China, Zhang et al. (2019) in the Asian region, Anwar et al. (2019) in 59 countries (low-middle-upper income) including Indonesia, Hundie and Daksa (2019) in Ethiopia, Kasman and Duman (2015) in EU countries, and Ozatac et al. (2017) in Turkey, shows that increasing urbanization will encourage increasingly massive use of energy which has an impact on environmental damage. The urbanization in Indonesia has grown significantly along with the rapid growth of the industry. In 1960, the percentage of the urban population only reached 14.6% of the total population, but in 2018, the percentage of urban population increased sharply to 55.3% of the total population (World Bank 2019). The study of Kurniawan and Managi (2018) concluded that urbanization in Indonesia has led to the increasing of coal consumption that rise caused rising of GHG emission.

The coefficient of the lag error correct method *ECM*_*t* − 1_has a negative and significant mark on both models. This shows that there is a long-term and short-term relationship of the variables. Lag value*ECM*_*t* − 1_the model is significant at the level of 1% with a coefficient of − 0.864736. This shows that any change in CO_2_ emissions from the short term to the long term will be corrected by 86.47% each period.

### Causality

**Fig. 2:**
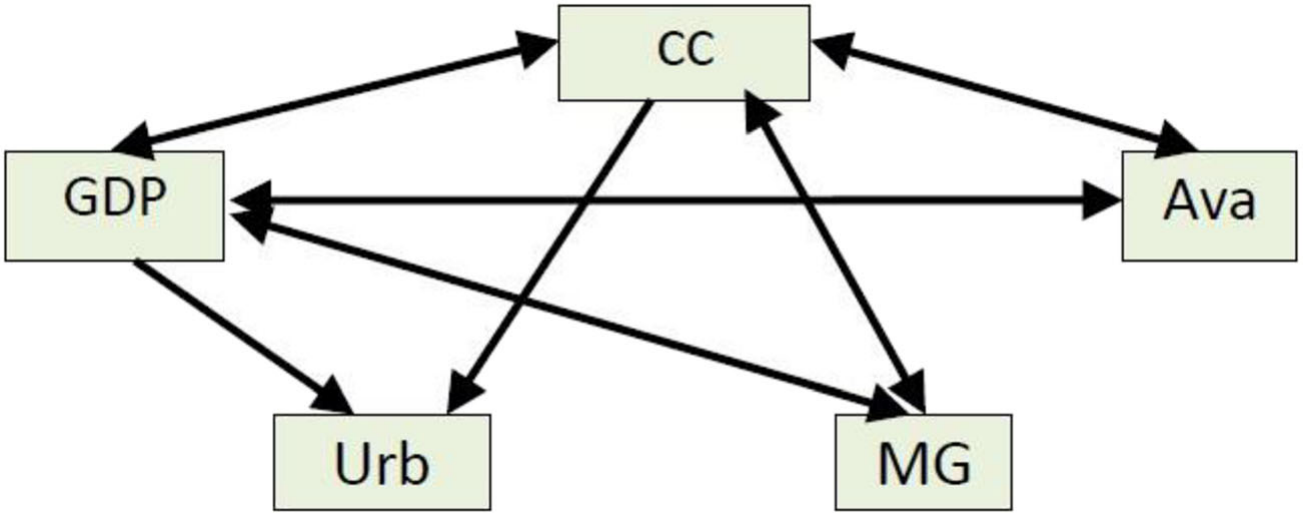
Result of causality of the model. Note:  : unidirectional causality,  : bidirectional causality

The existence of long-term and short-term causality is obtained from the Granger Causality test in Table 5. The results of the causality test in the bidirectional causality are confirmed between CC with GDP, CC with Ava, and CC with MG, GDP with Ava, and GDP with MG, whereas in unidirectional causality are confirmed on the variables CC with Urb and GDP with Urb. Long-run causality exists in models with dependent variables CC, GDP, Ava, and MG (Fig. 2).

**Table 5:** Long-run and short-run analysis

Regressor	Coefficient	Probability		Coefficient	Probability
Variable	Long run		Variable	Short run	
GDP	0.128824**	0.0167	∆GDP	0.128824***	0.0001
GDP^2^	− 3.13E−05***	0.0099	∆GDP^2^	− 3.13E−05***	0.0000
Ava	− 2.530118***	0.0015	∆Ava	− 2.530118***	0.0000
MG	1.688486**	0.0150	∆EO	1.688486***	0.0000
Urb	14.27803**	0.0481	∆IO	14.27803***	0.0000
Dumbreak1981	5.582291**	0.0101	∆Dumbreak1981	5.582291***	0.0003
C	34.25102	0.4383	C	34.25102***	0.0000
			ECM_*t*−1_	− 0.864736***	0.0000
R-squared	0.989480			0.963428	
Adj. R-squared	0.966821			0.921083	
F-statistic	43.66894***	0.0000		22.75136***	0.0000
LM test	*x*^2^ = 2.957299 (prob = 0.1112)				
Normality test	Jarque bera: 3.145424 (prob = 0.207482)				
Heteroskedasticity test (ARCH test)	*x*^2^ = 1.021041 (prob = 0.4109)				
Turning point	2856.61				

*,**,***10, 5, and 1% level of significance

The results of the causality test (Table 6) show the bidirectional causality between emissions and GDP in Indonesia is consistent with the research of Shahbaz et al. (2013). The bidirectional causality between GHG emissions and the agricultural sector has never been carried out before in Indonesia, but these results are consistent with the study of Anwar et al. (2019) in middle- and upper-income countries; Gokmenoglu and Taspinar (2018) in Pakistan; and Jebli and Youssef (2016) in Tunisia, while the bidirectional causality between GDP and the agricultural sector in Indonesia is consistent with research carried out by Nugraha and Osman (2018).

**Table 6:** VECM Granger Causality analysis

Dependent variable		Short-run causality					Long-run causality
	∆CC	∆GDP	∆GDP^2^	∆Ava	∆Mg	∆Urb	ECM_*t*−1_
∆CC	-	3.8006** (0.0371)	4.6179** (0.0154)	11.049*** (0.0004)	4.5003** (0.0168)	14.278** (0.0481)	− 0.8647*** (0.0036)
∆GDP	6.7301*** (0.0022)	-	0.0002*** (0.0000)	− 4.997** (0.0150)	20.108*** (0.0000)	3.7473** (0.0258)	− 0.1285*** (0.0019)
∆GDP^2^	9.0125*** (0.0046)	963.6*** (0.0000)	-	3.1022* (0.0890)	17.458*** (0.0005)	2.7074 (0.1075)	− 0.0027 (0.5410)
∆Ava	8.5091*** (0.0011)	3.5260** (0.0432)	2.8321* (0.0765)	-	2.0089 (0.1590)	2.4559 (0.1297)	− 0.6341** (0.0119)
∆Mg	2.7104* (0.0686)	3.9515** (0.0207)	4.0733** (0.0185)	1.7716 (0.1807)	-	− 0.0208 (0.7989)	− 0.2656* (0.0987)
∆Urb	1.575 (0.2191)	0.0003 (0.6409)	0.00000068 (0.6753)	0.0200 (0.1990)	− 0.0058 (0.6309)	-	− 0.0074 (0.1796)

*,**,***10, 5, and 1% level of significance

## Conclusion and policy recommendations

This study showed how the effects of economic growth, the agricultural sector, manufacturing, and urbanization are the causes of carbon emissions in the EKC hypothesis. The EKC hypothesis was researched and confirmed in this study. From the obtained results, it can be concluded that in the case of Indonesia, the turning points reach at 2057.89 USD/capita and therefore, the EKC turning point has been exceeded.

Empirical results in this study indicate that the variable gross domestic product, manufacturing value added, and urbanization play a role in increasing total carbon emissions in Indonesia, while the growth of the agricultural sector (agriculture value added) will reduce total carbon emissions in Indonesia. In agriculture and manufacturing sector emissions, economic growth and agriculture value added contributed positively to emissions. Although empirical tests show that the agricultural sector is negatively correlated to total emissions, however, data from (World Bank 2019) shows that agriculture contribution to GDP continues to decline. This causes increased carbon emissions from the agricultural sector in Indonesia. Besides that the causality test also shows that carbon emissions have bidirectional causality on economic growth, agriculture value added, and manufacturing value added.

Considering the results of this study, the government needs to pay attention to the agriculture and manufacturing sectors not only as the backbone of the national economy but also because these two sectors have great potential for environmental damage. In the agricultural sector, rice cultivation as the main emitter needs to get the most attention. Management of low-emission environmentally friendly rice fields is feasible. Some policies that can be carried out include agricultural campaigns with organic fertilizers, intermittent irrigation on lowland rice fields, and water-saving rice cultivation such as SRI (System of Rice Intensification). Studies from Ariani et al. (2018) and Setyanto et al. (2018) show that water-efficient rice cultivation and intermittent irrigation can reduce emissions by up to 45% without reducing the potential for harvests produced.

In the manufacturing and urbanization sector, the majority of GHG emissions generated come from the energy used, both for the production process, transportation, and for household energy needs. The government needs to encourage the manufacturing sector to use high technology in handling pollution, especially air pollution. The renewable energy use policy has long been carried out as mentioned in the “Results and discussion” section so that it can be improved. The use of renewable energy (geothermal, wind, solar power, micro-hydro) only reaches 5% of Indonesia’s potential, so the potential for development is still very large. In the transportation sector, the government can increase the use of biodiesel especially from palm oil–derived products, especially since Indonesia is currently the largest palm oil producer in the world.
